# The effect of ambient temperature on blood pressure of patients undergoing hemodialysis in the Pantanal-Brazil

**DOI:** 10.1016/j.heliyon.2021.e07348

**Published:** 2021-06-18

**Authors:** Shaiana Vilella Hartwig, Sandra de Souza Hacon, Beatriz Fátima Alves de Oliveira, Ludmilla da Silva Viana Jacobson, Raniere Flávio Viana Sousa, Eliane Ignotti

**Affiliations:** a State University of Mato Grosso, Brazil; b Oswaldo Cruz Foundation, Brazil; c Federal Fluminense University, Brazil

**Keywords:** Renal dialysis, Biometeorology, Bioclimatology, Temperature, Environmental health

## Abstract

The objective was to analyze the association of changes in pre-dialysis systolic and diastolic blood pressure with air temperature in a municipality in the Brazilian Pantanal, a tropical climate area. Longitudinal panel study, with analysis of mixed effects models of 133 hemodialysis patients in the city of Cáceres-Mato Grosso in 2014. Air temperature showed an inverse association with pre-dialysis systolic and diastolic blood pressure. With each increase of 1 °C in the mean air temperature, the pre-dialysis systolic blood pressure decreases -0.730mmHg (p ≤ 0.000) and the pre-dialysis diastolic blood pressure decreases -0.280mmHg (p ≤ 0.000). The estimated effect was greater for systolic blood pressure, but both pre-dialysis blood pressure measures are reduced with an increase in lag (up to two days), even when adjusted for relative air humidity. Air temperature is determinant for changes in pre-dialysis systolic and diastolic blood pressure in hemodialysis patients. The temperature effect was greater for systolic blood pressure than for diastolic blood pressure.

## 1 Introduction

The kidney is one of the organs responsible for controlling blood pressure. Hypertension can be the cause or the consequence of renal dysfunction, and the control of hypertension is essential for the prevention of the disease [1]. Air temperature is recognized as the main meteorological factor for altering blood pressure in humans [2, 3, 4, 5]. Among the groups most vulnerable to these changes (related to increased blood pressure at low temperatures) are the elderly, children, terminal patients and people with chronic diseases [6]. This group of people, when they need hemodialysis (HD), they have constant changes in blood pressure, especially Systemic Arterial Hypertension (SAH) [7].

With the initiation of hemodialysis treatment, some patients reverse SAH by removing excess fluids. However, some patients continue to present with hypertension [8], so much so that during hemodialysis, the main complication is related to changes in blood pressure, hypotension, and intradialytic hypertension [9, 10].

There are no limits on ideal and safe blood pressure values for patients on HD. On the other hand, the blood pressure values that are indicated for the general population, when applied to patients undergoing HD treatment, result in an increase in mortality in this group [11, 12].

Most studies show an inverse association between air temperature and blood pressure in hemodialysis patients [13, 14, 15, 16, 17, 18, 19, 20, 21, 22, 23, 24]. These studies were carried out predominantly in regions of Continental, Temperate, and Mediterranean climate, focusing on the analysis of seasonality.

The Intergovernmental Panel on Climate Change-IPCC [25] motivated studies on the impact of climate change on human health. In countries with a tropical climate, the prioritization of themes is related to the effects of climate change on neglected tropical diseases, especially those transmitted by vectors. These countries minimize the consequences of chronic non-communicable diseases, which will also be influenced by these climate changes [26, 27, 28].

Knowing that patients undergoing hemodialysis have uncontrolled blood pressure, it is relevant to understand the effects of variations in air temperature on the blood pressure of patients on hemodialysis residing in a tropical climate area such as the Brazilian Pantanal region. Quantifying the effect of air temperature on blood pressure changes will guide the adoption of practices to minimize blood pressure changes and their consequences for patients undergoing hemodialysis. The aim of this study was to analyze the association of changes in pre-dialysis systolic and diastolic blood pressure with air temperature in a municipality in the Pantanal of Mato Grosso.

## 2 Method

This research was designed as a longitudinal retrospective epidemiological study, with repeated measures, defined as a panel study. This analysis was carried out with chronic renal patients undergoing HD in 2014 in the city of Cáceres - Mato Grosso, Brazilian Pantanal. In terms of ethnicity, the population of the state of Mato Grosso is of mixed race, like most Brazilians. During the last census, 90% of the population self-classification black or “*parda*" brown color [29, 30].

For the study, data were collected from the medical records of 133 patients who signed the Free and Informed Consent Form. All patients were of legal age (over 18 years) and had been undergoing HD for more than three months. Data were collected in January 2014 at the only hemodialysis center in the region, located in the city of Cáceres. These studies are non-existent in Brazil due to the difficulty in obtaining data on patients undergoing treatment, so our study was limited to this group of patients from southwestern Mato Grosso.

The patients lived in 14 municipalities in the southwestern region of Mato Grosso, with a total population of 206,356 inhabitants [30]. Among the 133 participants, 69 (52%) lived in Cáceres, where there is a HD service. The municipalities in the region have the same climatic characteristics, according to the Köppen classification. Where in these regions, the climate is of the Tropical type, with two well-defined wet (November to March) and dry (May to September) periods. The Pantanal biome is located in the southwest region of the state of Mato Grosso. This biome is the largest low-lying tropical humid area, with 150,355 km2 of wetland on the planet [30]. To facilitate comparisons between the climatic characteristics, this research chose the Köppen classification as it is the most used in the world.

All patients had a prescription for hemodialysis three times a week. Eventually, there could be absences for some patients or extra sessions when requested by the medical team. From these patients, data were obtained on variables related to individual characteristics that do not change over time, including date of birth, sex, color/race, underlying disease, initial diagnosis, use of antihypertensive drugs. Information about the initial diagnosis is related to the diagnosis that led the individual to seek the nephrology service. This fact is not necessarily involved in starting hemodialysis, and in some cases, it may be the secondary diagnosis, as there is no such field in the medical record.

Monthly variations (hemoglobin measurement (g/dL)), free calcium (mg/dL), phosphorus measurement (mg/dL), glutamic-pyruvic transaminase - ALT (u/L), anti-HCV (reagent and not-reagent), or daily, such as: day of the hemodialysis session, blood pressure (mmHg) recorded (at the entrance, during and after the session) and pre-dialysis weight (kg). The patients were weighed at the entry and exit of the HD sessions using a digital scale, with weight checks in kg. In patients using a wheelchair and bedridden, no weighing was performed due to the lack of an appropriate weight scale (6.3% of the set of measures).

The dependent variables were pre-dialysis Systolic Blood Pressure (pre-SBP) and pre-dialysis Diastolic Blood Pressure (pre-DBP), whose measurements were taken before the hemodialysis procedure. Blood pressure measurements were performed by an indirect method, using the auscultatory technique with the use of an aneroid sphygmomanometer. This procedure was done in the armchair that patients underwent hemodialysis.

Adjustment variables include the use of antihypertensive drugs, diagnosis of disease (chronic end-stage disease, unspecified chronic insufficiency, acute nephrotic syndrome - proliferative glomerulonephritis, unspecified acute nephrotic syndrome, chronic nephrotic syndrome, and congenital kidney malformation), sex, age, and weight of entry, hemoglobin, calcium, TGP and anti-HCV.

For meteorological data, daily air temperature (maximum, minimum, and average) and relative humidity (maximum, minimum, and average) from January to December 2014 were considered. This information was obtained through the automatic meteorological station of the National Meteorological Institute (INMET) [31] installed in Cáceres (code - A941).

The association between air temperature and pre-SBP and preDBP was estimated using mixed effects models. These models allow both fixed and random effects; thus, they are appropriate for data analysis required in this study which in considering the independence between patients and the dependence in the time of the repeated measurements of each patient.

Adjusting the mixed effects model follows these steps:

(I) Adjustment of the time trend;
(II) Inclusion of variables related to individual characteristics fixed over time;
(III) Inclusion of variables that change over time (month and day);
(IV) Adjustment of the variance function of the random error;
(V) Adjustment of residual autocorrelation;
(VI) Evaluation of the effect of temperature and/or humidity.

In adjusting the trend, initially the variable that represents the observation day was centered on the midpoint of the study period. The trend was adjusted by a cubic spline, with a random effect on the parameters. The variance function of the random error was adjusted by the variables sex and age (categorized) and the residual autocorrelation by a common autocorrelation structure (AR(1)). Compound Symmetry structure was also evaluated, but AR(1) presented lower AIC.

The Likelihood-Ratio Test and the Akaike Information Criterion (AIC) were used to assess the entry of explanatory variables in the model and the quality of the adjustment. At the 5% significance level, the significant variables for adjusting the model were: use of antihypertensive drugs; disease diagnosis (chronic end-stage disease, unspecified chronic insufficiency, acute nephrotic syndrome - proliferative glomerulonephritis, unspecified acute nephrotic syndrome, chronic nephrotic syndrome, congenital kidney malformation), sex, age, weight of entry (with random effect in the parameter), hemoglobin, calcium, ALT, anti-HCV and relative humidity to assess the effect of air temperature. To adjust the meteorological variables, lag0 and lag1 measurements were used. For the main exposure variables, lag0 to lag3 and Arithmetic Moving Averages (MMA) were considered for the calculations. In order to estimate the cumulative effect of exposure to temperature, the Polynomial Distributed Lag Model (PDLM) technique was also considered. In this study, quadratic polynomials of the effects lagged up to 3 days were calculated. The data were analyzed in the R program (version 3.3.5) [32] through the *nlme* library [33].

The study was approved by the Research Ethics Committee of the State University of Mato Grosso, and Certificate of Presentation for Ethical Appreciation (CAAE): 49487815.0.0000.5166, in November 2015.

## 3 Results

**Figure 1:**
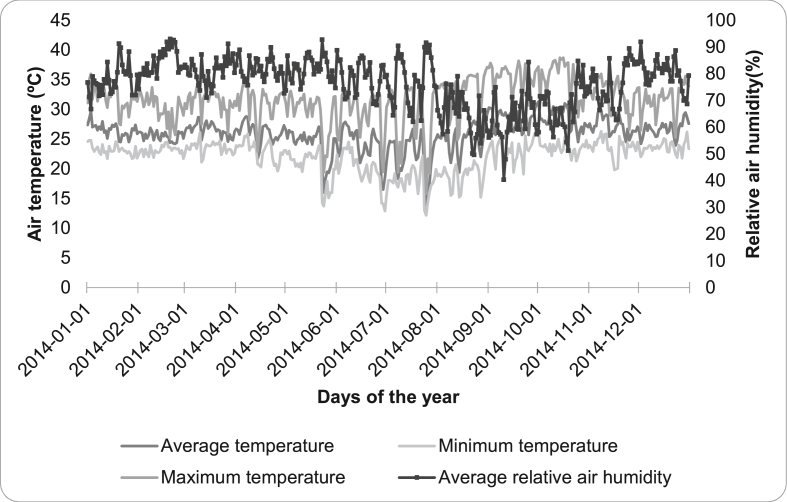
Daily distribution of the maximum, average and minimum air temperature (°C) and the average relative air humidity throughout 2014, Cáceres-MT.

In 2014, the average air temperature in Cáceres was 25.9 °C. The average maximum temperature was 31.7 °C, and the average minimum temperature was 21.7 °C. The records of minimum temperatures were concentrated between June and August, which registered 12.2 °C as the lowest temperature in this period. Regarding the maximum temperature, it increases from August, with a higher record of 38.7 °C in October. The average relative air humidity in the period was 76.2%, reaching a maximum of 92.8% in May and a minimum of 22% in September. The period of lowest record of relative humidity was from August to October (Figure 1).

**Figure 2:**
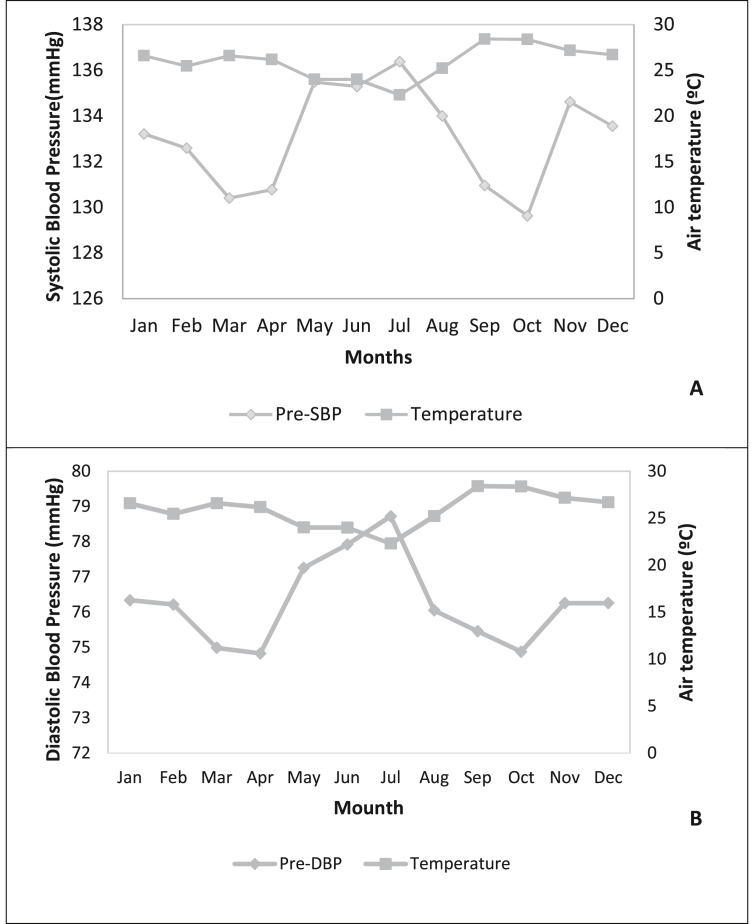
Mean variations in pre-dialysis systolic (A) and diastolic (B) blood pressure and average air temperature in the months of 2014, Cáceres-MT. Pre-SBP: pre-dialysis systolic blood pressure. Pre-DBP: pre-dialysis diastolic blood pressure.

An inverse relationship is observed between air temperature, and pre-SBP, and pre-DBP. The months of May, June, and July were the months with the lowest temperature averages in the year and also the months whose mean systolic and diastolic blood pressure was highest (Figure 2).

Most patients were men (61%), with a mean age of 55 (SD14.9) years. The mean pre-SBP was 133 mmHg (60–240 mmHg), the mean pre- DBP was 76.2 mmHg (30–150 mmHg) and the mean pre-dialysis weight was 67 kg (36–110 kg). For monthly laboratory tests, hemoglobin averaged 10.27g/dl (SD 2.05), hematocrit was 30.7% (SD 5.91), calcium dosage of 4.78mg/dl (SD 0.35), the ALT of 23.53U/l (SD 21.19) and 5 (3.4%) patients had the reagent Anti-HCV (Table 1).

**Table 1:** Descriptive statistics of meteorological variables and fixed, monthly and daily variables of patients undergoing hemodialysis in 2014, Cáceres-MT.

Variables	N	*Missing*	Average (SD)	Minimum	Percentile					Maximum
					10	25	50	75	90	
Temperature (°C)	365	0	25.92 (2.65)	12.2	22.6	24.8	26.2	27.4	28.9	37.8
Humidity (%)	365	0	76.20 (9.45)	22.0	62.0	71.0	77.9	83.2	86.7	100
Age (years)	133	0	54.93 (14.91)	21	33.4	46	56	65	74	92
Men	133	0	60.9%							
Pre-SBP (mmHg)	17.072	62	133.01 (24.7)	60	100	120	130	150	160	240
Pre-DBP (mmHg)	17.072	62	76.28 (13.36)	30	60	70	80	80	90	150
Pre-dialysis weight (kg)	16.127	1.014	66.76 (13.2)	36.0	50.4	57.6	66.1	75.7	82.5	110
Hemoglobin (g/dl)	1.589	7	10.27 (2.05)	3.4	7.7	8.9	10.2	11.6	12.8	28.8
Hematocrit (g/dl)	1.589	7	30.7 (5.91)	9.30	23.0	26.8	30.8	34.8	38.5	48.4
Calcium (mg/dl)	1.586	10	4.78 (0.35)	1.0	4.3	4.5	4.8	5.0	5.1	6.6
ALT (U/l)	1.583	13	23.53 (21.19)	7	10	12	18	26	43	262
Anti-HCV (reagent)	1.596	0	3.4%							

SBP: systolic blood pressure; DBP: diastolic blood pressure; ALT: glutamic-pyruvic transaminase.

The models of basic mixed effects for systolic and diastolic blood pressure, without the inclusion of meteorological variables were analyzed. The use of antihypertensive drugs was associated with an increase in the mean pre- SBP of 8.45 mmHg (p = 0.017). The diagnosis of end-stage renal disease was associated with an increase of 41.13 mmHg (p = 0.013) in pre-dialysis SBP, as well as the diagnosis of unspecified chronic renal failure with a positive association of 39.26 mmHg (p = 0.018). Pre-dialysis weight showed an association of 0.53mmHg (p ≤ 0,000) in the increase in pre- SBP; in which, for a gain of 1kg there is an increase in mean SBP of 0.53mmHg. With regard to laboratory tests, it appears that with each increase of a unit in hemoglobin (HB) there is an increase of 0.48mmHg (p = 0.002) in the mean of the SBP; and the increase of one unit in the ALT is associated with an increase of 0.02mmHg (p = 0.021) in the mean of the SBP (Table 2).

**Table 2:** Basic model without meteorological variables, for estimates of changes in pre-dialysis systolic and diastolic blood pressure, Cáceres-MT-2014.

Systolic blood pressure				
Variables	Estimate for SBP	p-value (test t)	IC 95%	
			Bottom	Higher
Antihypertensive use	**8,45**	*0,017*	1,57	15,33
Diagnosis of unspecified acute nephrotic syndrome	33,38	0,137	-10,39	77,15
Diagnosis of chronic nephrotic syndrome	30,05	0,217	-17,40	77,50
Diagnosis of end-stage renal disease	**41,13**	*0,013*	9,08	73,18
Diagnosis of unspecified end-stage renal disease	**39,26**	*0,018*	7,04	71,48
Diagnosis of other specified congenital malformations of the kidney	38,67	0,086	-5,21	82,55
Gender (female)	3,50	0,281	-2,83	9,83
Age (>60 years old)	0,54	0,863	-5,59	6,67
Weight of entry	**0,53**	*0,000*	0,39	0,67
Hemoglobin	**0,48**	*0,002*	0,19	0,77
Calcium	0,34	0,639	-1,07	1,75
ALT	**0,02**	*0,021*	0,00	0,04
**Diastolic blood pressure**				
Variables	Estimate for DBP	p-value (test t)	IC 95%	
			Bottom	Higher
Diagnosis of unspecified acute nephrotic syndrome	**27,58**	*0,005*	8,35	46,81
Diagnosis of chronic nephrotic syndrome	**36,73**	*0,000*	15,88	57,58
Diagnosis of end-stage renal disease	**33,65**	*0,000*	18,81	48,49
Diagnosis of unspecified end-stage renal disease	**34,32**	*0,000*	19,03	49,61
Diagnosis of other specified congenital malformations of the kidney	**20,02**	*0,048*	0,32	39,72
Age	**-0,36**	*0,000*	-0,48	-0,24
Gender (female)	-0,71	0,584	-3,26	1,84
Idade (>60 years old)	1,77	0,368	-2,07	5,61
Weight of entry	**0,14**	*0,000*	0,08	0,20
Hematocrit	**0,16**	*0,000*	0,10	0,22
ALT	0,00	0,312	0,00	0,00
Anti-HCV Reagent	**10,85**	*0,007*	3,09	18,61

ALT: glutamic-pyruvic transaminase.

As for clinical variables, it is observed that the diagnosis of unspecified acute nephrotic syndrome, chronic nephrotic syndrome, unspecified nephrotic syndrome, congenital kidney malformation, sex, age and calcium concentration did not show statistically significant associations with pre- SBP, but were kept in the model due to the quality of the adjustments.

For pre-DBP, it is observed that the diagnoses are positively associated, with the unspecified acute nephrotic syndrome associated with an increase of 27.58mmHg (p < 0.005) of the pressure; the diagnosis of chronic nephrotic syndrome at an increase of 36.73mmHg (p ≤ 0,000) in pressure; the diagnosis of end-stage renal disease with an increase of 33.65mmHg (p ≤ 0,000) in pressure. And the diagnosis of other congenital malformations specific to the kidney with an increase of 20.02mmHg (p = 0.048) in pressure.

The age of patients had an inverse association of -0.36mmHg (p ≤ 0.000) with pressure. Thus, with each increase of one year of age, the mean pre-DBP decreased by -0.36mmHg. Pre-dialysis weight showed an association of 0.14 mmHg (p ≤ 0,000) in the increase in pre- DBP. Thus, for every 1 kg gain by the patient, there was an increase in the pre- DBP. The results of laboratory tests of hematocrit and anti-HCV reagent also showed a positive association with pre-SBP. With each increase of one unit in hematocrit (HT) there was an increase of 0.16mmHg (p ≤ 0,000) in the mean of the DBP. And anti-HCV reactive patients are associated with an increase of 10.85mmHg (p = 0.007) in the mean DBP. Thus, the patient having hepatitis C increases the pre- DBP.

The clinical variables gender, age and ALT did not show statistically significant associations with pre- DBP, but were maintained in the model for better adjustment (Table 2).

After adjusting the basic model, the variables temperature and relative humidity were included for the analysis. To assess the effect of temperature, the variable average relative humidity on the current day (lag 0) or lagged one day (lag 1) was included in the model as a covariate. The final model included the basic model, relative humidity on the current day and temperature (lag 0, lag 1, lag 2, lag3 or moving average for lags 1 and 2) (Table 3). For SBP the model adjusted for humidity in lag 0 shows that with each increase of 1 °C in the average temperature there is a reduction in the pre- SBP on the day of the session (lag 0 temperature) of -0.730mmHg (p ≤ 0.000); Statistically significant results were verified for temperature in lag 1, 2 and 3 and the moving average of two days before the hemodialysis session.

**Table 3:** Effects of temperature adjusted by the variables included in the basic model and relative humidity (lag 0) for pre-dialysis systolic and diastolic blood pressure.

Systolic blood pressure						
	Temperature	Estimate for SBP	CI 95%		p-value	AIC
			Bottom	Higher		
Basic model + Humidity lag 0	Lag 0	-0,730	-0,907	-0,552	*0,000*	136188,6
	Lag 1	-0,462	-0,604	-0,319	*0,000*	135798,6
	Lag 2	-0,220	-0,355	-0,086	*0,001*	135379,4
	Lag 3	-0,278	-0,408	-0,148	*0,000*	134842,1
	Moving average lag 1e2	-0,398	-0,550	-0,247	*0,000*	135362,6
**Diastolic blood pressure**						
	Temperature	Estimate for DBP	CI 95%		p-value	AIC
			Bottom	Higher		
Basic model + Humidity lag 0	Lag 0	-0,281	-0,382	-0,181	*0,000*	118775,9
	Lag 1	-0,242	-0,323	-0,160	*0,000*	118412,5
	Lag 2	-0,131	-0,208	-0,054	0,008	118032,7
	Lag 3	-0,110	-0,184	-0,036	*0,004*	117564,3
	Moving average lag 1e2	-0,217	-0,303	-0,131	*0,000*	118019,3

Italic value indicates the significant values <0.05

For DBP, when adjusted for relative air humidity at lag 0, with a 1 °C increase in the average temperature, a reduction in pre-dialysis DBP was observed on the day of the hemodialysis session (lag 0) (-0.281mmHg; p ≤ 0,000); the day before the hemodialysis session (lag 1) (-0.242mmHg; p ≤ 0,000); in the two days preceding the hemodialysis session (lag 2) (-0.131mmHg; p = 0.008); in the three days preceding the hemodialysis session (lag 3) (-0.110 mmHg; p = 0.004) and in the two-day moving average (-0.217 mmHg; p ≤ 0,000) (Table 3).

By the approach of the distributed lag polynomial models, the global effect of exposure to temperature in lags 1, 2, 3 and current day was significant for both systolic and diastolic blood pressure, with a reduction in pre-SBP of 0.956mmHg (95% CI: - 1,171mmHg; -0,742mmHg) and reduction in the pre-DBP of 0,385mmHg (95% CI: -0,506 mmHg; -0,265 mmHg) for each 1 °C increase in the average temperature (Table 4). The final model included the basic model, relative humidity in the current day and temperature with the structure of the PDLM.

**Table 4:** Effects of temperature estimated by the approach of polynomial models of distributed lag for pre- systolic and diastolic blood pressure.

Systolic blood pressure			
Temperature	Estimate for SBP	CI 95%	
		Bottom	Higher
Lag 0	-0,698	-0,887	-0,509
Lag 1	-0,052	-0,158	0,055
Lag 2	0,084	-0,023	0,191
Lag 3	-0,291	-0,444	-0,137
Global	-0,956	-1,171	-0,742
**Diastolic blood pressure**			
Temperature	Estimate for DBP	CI 95%	
		Bottom	Higher
Lag 0	-0,281	-0,336	-0,116
Lag 1	-0,084	-0,147	-0,021
Lag 2	-0,026	-0,089	0,038
Lag 3	-0,050	-0,140	0,040
Global	-0,385	-0,506	-0,265

## 4 Discussion

This is the first study to quantify the changes in SBP and DBP of patients undergoing hemodialysis about temperature variations in a tropical climate area. The analysis shows that the greater the daily variations in temperature, the greater the changes in systolic and diastolic blood pressure during the pre-dialysis period. The analysis shows that the greater the daily variations in temperature, the greater the changes in pre-dialysis systolic and diastolic blood pressure.

Although the scientific literature has demonstrated the inverse relationship between air temperature and blood pressure [34] for patients who are not undergoing hemodialysis [3, 35, 36, 37, 38] and patients undergoing hemodialysis [13, 14, 16, 17, 18, 19, 20, 21, 22, 23, 24, 39, 40], the quantification of this relationship using mixed-effect models adjusted in a longitudinal study has not been demonstrated for hemodialysis patients in Brazil.

For the general population, a study conducted with data from 16 countries (Europe and North America), it was found that the increase of 1 °C in the average external temperature decreased -0.19 mmHg (95% PI: - 0.26; - 0.11 mmHg) of the SBP [41]. In a meta-analysis review with adults, patients with diseases related to cardiovascular problems, such as hypertensive, diabetics, and patients with myocardial ischemia, presented changes in blood pressure greater than the general population, with an increase of 1 °C in the mean temperature. The SBP decreases - 0.38 mmHg (0.18–0.58), a difference of 0.12 mmHg in relation to the general population [42].

In the present study, patients undergoing hemodialysis, the relationship found was even more evident, implying a reduction of -0.730mmHg (p ≤ 0,000) in SBP and -0.280mmHg (p ≤ 0,000) in pre-dialysis DBP. Although the methods used are different, this difference can indicate the biological vulnerability to temperature changes in patients on hemodialysis when compared to the general population and also to patients with other chronic diseases.

Variations in SBP and DBP influenced by air temperature can further impair the evolution of patients undergoing hemodialysis whose risk of cardiovascular mortality in these people was associated with either a reduction or an increase in blood pressure [43, 44]. Thus, both episodes of hypotension and hypertension can contribute to an increased risk of mortality from cardiovascular diseases in patients undergoing hemodialysis.

Although the link between air temperature and blood pressure has been established, the mechanisms underlying this relationship have not been fully understood to date, and there are likely to be multiple [6, 23, 38, 45, 46].

When the body is exposed to low temperatures, the first physiological response will be peripheral vasoconstriction., which will reduce thermal conduction through the skin and trigger tremors and reduce diuresis. This response mediated by sympathetic activation increases heart rate, renin-angiotensin activity, and aldosterone levels, which raise blood pressure [47, 48, 49].

Other physiological responses involved in the increase in blood pressure due to the decrease in temperature are platelet activation [50], erythrocyte deformability, blood viscosity [43], the cumulative increase in the levels of blood inflammatory markers [51], activation of type L calcium channels [52], and oxidative stress [53, 54]. These indicators show the difficulty of these patients in presenting physiological resilience to reduce the temperature.

The response to heat (temperature increased) is more systemic when compared to cold (temperature decrease), whose first reaction of the organism is peripheral vasodilation and sweating to lower the core temperature [49]. With the loss of peripheral resistance, sympathetic activation prevents a critical loss of blood pressure through a compensatory increase in heart rate and systolic volume. The mechanism of sweat, the simultaneous loss of salt and the reduction of blood volume are also involved [46]. There are also water losses due to perspiration, and breathing that are relevant in patients undergoing hemodialysis [16, 55, 56].

Pre-SBP is generally used for the management of hypertension in patients undergoing hemodialysis [46]. The association between temperature and blood pressure was statistically significant only for pre-DSP and SBP. The relationship between temperature and intra-dialysis and post-dialysis blood pressure has also been studied in some patients undergoing hemodialysis, but no association was found in this group of people [15, 23, 54]. In this study, this relationship was explored and the results corroborated with those mentioned above.

Elucidating the temperature response on the pre-SBP and DBP, a moment when the influence of the external air temperature is higher because the patient has recently been in the environment of the session, which is air-conditioned.

In the present study, pre- SBP and DBP were associated with clinical and laboratory variables. Both pressures were positively associated with chronic kidney disease diagnoses. The influence of diagnoses on blood pressure can be explained by the pathophysiological mechanism of the disease [55].

Pre-dialysis weight was positively associated with pre SBP and DBP, and the increase in blood pressure is related to fluid overload, which is a consequence of CKD [16, 46]. It is important to remember that this variable can be controlled by the correct intake of fluids by patients in the interdialytic period [16, 24, 44, 57]. The increase in hemoglobin and hematocrit, with the increase in SBP and DBP, is related to the increase in blood viscosity [46, 58].

The relationship between increased ALT concentration and SBP has not yet been described in the literature, and therefore requires further clarification. There were also no studies that related the association of increased DBP in patients with hepatitis C in hemodialysis. ALT is used as a marker of liver function, and elevation is indicative for screening for infection by hepatitis B and C viruses [59, 60]. These associations between ALT and SBP and the positivity for hepatitis C with increased DBP need to be further studied.

Pre-SBP was positively associated with hypertensive patients. The relationship between hypertension and chronic kidney disease can be related to the cause or consequence of the disease [1]. In Brazil, 80–90% of hemodialysis patients are hypertensive [60, 61]. Studies on the number of drugs for the treatment of blood pressure in patients undergoing hemodialysis also showed that the increase in antihypertensive drugs is not able to control blood pressure. Demonstrating the difficulty in controlling pressure in these patients and the relationship of blood pressure with other variables [16, 46, 55, 56, 62].

The inverse association between age and DBP was contrary to the findings of other studies, whose association found was positive for patients undergoing hemodialysis [3, 62].

Knowing the association between air temperature and blood pressure, it is evident that we must analyze weather conditions, focusing on local temperature and the possible impacts of climate change for this group of patients, who are vulnerable to changes in blood pressure as the rise in air temperature.

The projections for the Pantanal region in the scenario projected by the IPCC [25] will be for a 1 °C increase in temperature and a decrease of between 5% and 15% in rainfall patterns by 2040 [63]. For projections of the heat stress index (WBGT) - an indicator of thermal stress that combines temperature, humidity, wind speed and radiation - the projection of the maximum scenario (average of the maximum) until 2040, is estimated for the studied region thermal stress between September and November of 31.5 °C. It is the limit considered alert for physiological adaptation, depending on the metabolic expenditure [64]. These projections may indicate a scenario of difficult adaptation of patients on hemodialysis to this new climatic condition.

The increase in temperature will lower blood pressure, but the increase in heat associated with a decrease in the relative humidity of the air increases the feeling of thirst, which can increase fluid intake [33] and blood pressure control for patients on hemodialysis should be more constant and rigorous.

The missing data represented 12.7% of the records, a situation for which we judged the data to be robust. As a limitation of the study, the meteorological data and the blood pressure measurements were collected in the municipality of treatment. Despite this limitation, it is noteworthy that approximately 52% of patients live in the municipality of treatment and 48% in neighboring municipalities. This region, as described in the materials and methods section, presents the same climatic characteristics, according to the Köppen classification.

However, this is the municipality that concentrates half of the study population. Although the adjustments of the models may have been influenced, the observations in lag 0 reinforce our findings. It was impossible to perform stratified analyzes by gender and age group due to the number of participants. The temperature data series refers to meteorological data, as it covers only one year. However, the results are extremely relevant to public health due to the clinical implications of this group's extreme biological vulnerability.

Our findings show the relevance of the change in air temperature in the diagnosis and treatment of these patients. Health professionals must be prepared for the adjustments of medications, liquid control conducts, and temperature changes [23, 24, 65]. Thus, it is important to protect against cold, in times of lower temperatures, such as wearing clothes that promote heating in an external environment, reducing the effect of cold on blood pressure [41]. It is also necessary that measures to control the increase in air temperature are implemented so that climate changes are less felt and thus have less impact on health.

Due to the difficulties in reaching goals and controlling blood pressure in patients undergoing hemodialysis [66, 67], the findings of this study point to the need to consider meteorological variations, especially air temperature, in clinical guidelines and protocols, as well as in quality of life campaigns. As changes in weather variations are seasonal and predictable, recommendations can assist in controlling by minimizing variations in blood pressure.

Longitudinal epidemiological studies of the effects of air temperature and other meteorological factors on health - in addition to the direct impacts of meteorological variables - will provide a more accurate measure of health impacts and will help in planning and allocating resources to priorities.

## 5 Conclusion

It is concluded that air temperature is inversely associated with pre-dialysis systolic and diastolic blood pressure in patients undergoing hemodialysis treatment. The influence of temperature variation on systolic blood pressure is greater than diastolic and the acute effect is greater than the cumulative effect.

## Declarations

### Author contribution statement

Shaiana Vilella Hartwig and Eliane Ignotti: Conceived and designed the experiments; Performed the experiments; Analyzed and interpreted the data; Contributed reagents, materials, analysis tools or data; Wrote the paper.

Sandra de Souza Hacon and Raniere Flávio Viana Sousa: Contributed reagents, materials, analysis tools or data; Wrote the paper.

Beatriz Fátima Alves de Oliveira and Ludmilla da Silva Viana Jacobson: Performed the experiments; Analyzed and interpreted the data; Wrote the paper.

### Funding statement

This work was supported by the 10.13039/501100002322Coordenação de Aperfeiçoamento de Pessoal de Nível Superior - Brasil (CAPES) - Finance Code 001 and the 10.13039/501100005286Research Support Foundation of the State of Mato Grosso - Brasil (FAPEMAT), by providing the research grant. And it had a partnership with Rede Clima - Brasil, nº 550022/2014-7 and FINEP/ Climate Network (01.13.0353.00).

### Data availability statement

The authors are unable or have chosen not to specify which data has been used.

### Declaration of interests statement

The authors declare no conflict of interest.

### Additional information

No additional information is available for this paper.

## References

[1] Daugirdas J.T., Blake P.G., Ing T.S. (2016). Manual de Diálise.

[2] Brennan P.J., Greenberg G., Miall W.E., Thompson S.G. (1982). Seasonal variation in arterial blood pressure. Br. Med. J..

[3] Lewington S., Li L., Sherliker P. (2012). Seasonal variation in blood pressure and its relationship with outdoor temperature in 10 diverse regions of China: the China Kadoorie Biobank. J. Hypertens..

[4] Gronlund C.J., Sheppard L., Adar S.D., O’Neill M.S., Auchincloss A., Madrigano J., Diez Roux A.V. (2018 Nov). Vulnerability to the cardiovascular effects of ambient heat in six U.S. Cities. Epidemiology.

[5] Zhao H., Jivraj S., Moody A. (2019 March). “My blood pressure is low today, do you have the heating on?” the association between indoor temperature and blood pressure. J. Hypertens..

[6] Liu C., Yavar Z., Sun Q. (2015). Cardiovascular response to thermoregulatory challenges. Am. J. Physiol. Heart Circ. Physiol..

[7] Lee S., Oh H.J., Lee E.K., Lee O. (2017 June). Blood pressure control during chronic kidney disease progression. Am. J. Hypertens..

[8] Dinesh K., Kunaparaju S., Cape K., Flythe J., Feldman H., Brunelli S. (2011). A model of systolic blood pressure during the course of dialysis and clinical factors associated with various blood pressure behaviors. Am. J. Kidney Dis..

[9] Park J., Rhee C., Sim J. (2013). A comparative effectiveness research study of the change in blood pressure during hemodialysis treatment and survival. Kidney Int..

[10] Flythe J., Xue H., Lynch K., Curhan G., Brunelli S. (2015). Association of mortality risk with various definitions of intradialytic hypotension. J. Am. Soc. Nephrol..

[11] Hamrahian S.M., Falkner B. (2016). Hypertension in Chronic Kidney Disease. Adv Exp Med Biol - Advances in Internal Medicine.

[12] Turner J.M., Peixoto A.J. (2017). Blood pressure targets for hemodialysis patients. Kidney Int..

[13] Tozama M., Iseki K., Iseki C. (1999). Seasonal blood pressure and body weight variation in patients on chronic hemodialysis. Am. J. Nephrol..

[14] Spósito M., Nieto F.J., Ventura J.E. (2000). Seasonal variations of blood pressure and overhydration in patiens on chronic hemodialysis. Am. J. Kidney Dis..

[15] Cheung A.K., Yan G., Greene T. (2002). Seasonal variations in clinical and laboratory variables among chronic hemodialysis patiens. J. Am. Soc. Nephorol..

[16] Argiles A., Ronan L.M.F., Servel O., Chong G., Kerr P.G., Mourad G. (2004). Seasonal modifications in blood pressure are mainly related to interdialytic body weight gain in dialysis patients. Kidney Int..

[17] Argani H., Javanshir M. (2004). Seasonal variations of blood pressure in hemodialysis and renal transplant recipients. Transplant. Proc..

[18] Hwang J.C., Wang C.T., Chien C.C. (2007). Effect of climatic temperature on fluid gain in hemodialysis patients with different degrees of overhydration. Blood Purif..

[19] Takenaka T., Kojima E., Keita T. (2010). Seasonal variations of daily changes in blood pressure among hypertensive patients with end-stage renal diseases. Clin. Exp. Hypertens..

[20] Usvyat L.A. (2011). Seasonal variations in mortality, clinical, and laboratory parameters in hemodialysis patients: a 5-year cohort study. Clin. J. Am. Soc. Nephrol..

[21] Guinsburg A.M. (2015). Seasonal variations in mortality and clinical indicators in international hemodialysis populations from the MONDO registry. BMC Nephrol..

[22] Broers N.J.H., Usvyat L.A., Marcelli D. (2014). Season affects body composition and estimation of fluid overload in haemodialysis patients: variations in body composition; a survey from the European MONDO database. Nephrol. Dial. Transplant..

[23] Duranton F. (2018). Blood pressure seasonality in hemodialysis patients from five European cities of different latitudes. Kidney Blood Pres. Res..

[24] Duranton F. (2018). Geographical variations in blood pressure level and seasonality in hemodialysis patients. Hypertension.

[25] Intergovernmental Panel on Climate Change IPCC (2007). Climate Change 2007: the Physical Science Basis, Summary for Policy Makers.

[26] (2009). Brasil. Lei n. 12.187, de 29 de dezembro de 2009. Institui a Política Nacional sobre Mudança do Clima - PNMC e dá outras providências. Diário Oficial [da] República Federativa do Brasil, Poder Executivo, Brasília, DF. Edição Extra, Seção 1: 109. Brasil.

[27] Brasil. Ministério da Saúde (2013). Plano Setorial da Saúde para Mitigação e Adaptação à Mudança do Clima. Brasília.

[28] Brasil. Ministério do Meio Ambiente. Grupo Executivo do Comitê Interministerial de Mudança do Clima (2015). Plano Nacional de Adaptação das Mudanças climáticas. Brasília.

[29] Sidra. Sistema IBGE de Recuperação Automática. Censo demográfico. http://sidra.ibge.gov.br/tabela/3175.

[30] Instituto Brasileiro de Geografia e Estatística. Cidades 2019. http://ibge.gov.br/cidadesat/xtras/perfil.php?lang=&codmun=510250&search=mato-grosso.caceres.

[31] Instituto Nacional de Meteorologia Estações automáticas. http://www.inmet.gov.br/portal/index.php?r=estacoes/estacoesAutomaticas.

[32] R Development Core Team (2016). R: A Language and Environment for Statistical Computing.

[33] Pinheiro J., Bates D., Debroy S., Sarkar D., Development Core Team (2012). Nlme: Linear and Nonlinear Mixed Effects Models. R Package Version 3.1-104.

[34] Guyton A.C., Hall J.E. (2017). Guyton e Hall: Tratado de Fisiologia Médica.

[35] Modesti P.A., Rapi S., Bamoshmoosh M. (2012). Impact of one or two visits strategy on hypertension burden estimation in HYDY, a population-based cross sectional study: implications for healthcare resource allocation decisionmaking. BMJ Open.

[36] Aubinière-Robb L., Jeemon P., Hastie C.E. (2013). Blood pressure response to patterns of weather fluctuations and effect on mortality. Hypertension.

[37] Modesti P.A. (2013). Season, temperature and blood pressure: a complex interaction. Eur. J. Intern. Med..

[38] Su D., Du H., Zhang X. (2014). Season and outdoor temperature in relation to detection and control of hypertension in a large rural Chinese population. Int. J. Epidemiol..

[39] Argiles A., Mourad G., Mion C. (1998). Seasonal changes in blood pressure in patients with end stage renal disease treated with hemodialysis. N. Engl. J. Med..

[40] De Castro M.M.C., Mion Junior D., Marcondes M. (1998). Seasonal variation of blood pressure in maintenance hemodialysis. Rev. Paul. Med..

[41] Barnett A.G., Sans S., Salomaa V., Kuulasmaa K., Dobson A.J. (2007). The effect of temperature on systolic blood pressure. Blood Pres. Monit..

[42] Wang Q., Li C., Guo Y., Barnett A.G., Tong S., Phung D. (2017). Environmental ambient temperature and blood pressure in adults: a systematic review and meta-analysis. Sci. Total Environ..

[43] Robinson B.M., Tong L., Zhang J., Wolfe R.A., Goodkin D.A., Greenwood R.N., Kerr P.G., Morgenstern H., Li Y., Pisoni R.L., Saran R., Tentori F., Akizawa T., Fukuhara S., Port F.K. (2012). Blood pressure levels and mortality risk among hemodialysis patients in the Dialysis Outcomes and Practice Patterns Study. Kidney Int..

[44] Duranton F., Duny Y., Szwarc I., Deleuze S., Rouanet C., Selcer I., Maurice F., Rivory J.P., Servel M.F., Jover B., Brunet P., Daurès J.P., Argilés À. (2016). Early changes in body weight and blood pressure are associated with mortality in incident dialysis patients. Clin. Kidney J..

[45] Cuspidi C., Ochoa J.E., Parati G. (2012). Seasonal variations in blood pressure: a complex phenomenon. J. Hypertens..

[46] Shafi T. (2014). Predialysis systolic BP variability and outcomes in hemodialysis patients. J. Am. Soc. Nephrol..

[47] Sun Z., Cade R., Zhang Z., Alouidor J., Van H. (2003). Angiotensinogen gene knockout delays and attenuates cold-induced hypertension. Hypertension.

[48] Hong Y.C., Kim H., Lim Y.H., Yoon H.J., Kwon Y.M., Park M. (2013). Identification of ras genotypes that modulate blood pressure change by outdoor temperature. Hypertension.

[49] Castellani J.W., Young A.J. (2016). Human physiological responses to cold exposure: acute responses and acclimatization to prolonged exposure. Auton. Neurosci..

[50] Zhang J.N. (2004). Effects of low temperature on shear-induced platelet aggregation and activation. J. Trauma.

[51] Halonen J.I., Zanobetti A., Sparrow D., Vokonas P.S., Schwartz J. (2010). Associations between outdoor temperature and markers of inflammation: a cohort study. Environ. Health.

[52] Saad W.A., Guarda I.F., Camargo L.A., Santos T.A. (2008). Nitric oxide and l-type calcium channel influences the changes in arterial blood pressure and heart rate induced by central Angiotensin II. Behav. Brain Funct..

[53] Gwathmey T.M., Pendergrass K.D., Reid S.D., Rose J.C., Diz D.I., Chappell M.C. (2010). Angiotensin-(1–7)-angiotensin-converting enzyme 2 attenuates reactive oxygen species formation to angiotensin ii within the cell nucleus. Hypertension.

[54] Martarelli D., Cocchioni M., Scuri S., Spataro A., Pompei P. (2011). Cold exposure increases exercise-induced oxidative stress. J. Sports Med. Phys. Fit..

[55] (2007). National kidney foundation KDOQI clinical practice guideline and clinical practice recommendations for anemia in chronic kidney disease: 2007 update of hemoglobin target. Am. J. Kidney Dis..

[56] Wystrychowski G., Wystrychowski W., Zukowska-Szczechowska E., Tomaszewski M., Grzeszczak W. (2005). Selected climatic variables and blood pressure in Central European patients with chronic renal failure on haemodialysis treatment. Blood Pres..

[57] Kovacic V., Kovacic V. (2004). Seasonal variations of clinical and biochemical parameters in chronic haemodialysis. Ann. Acad. Med. Singapore.

[58] Loutradis C., Sarafidis P.A., Ekart R., Papadopoulos C., Sachpekidis V., Alexandrou M.E., Zoccali C. (2019). The effect of dry-weight reduction guided by lung ultrasound on ambulatory blood pressure in hemodialysis patients: a randomized controlled trial. Kidney Int..

[59] Atsma F., Veldhuizen I., de Kort W., van Kraaij M., Pasker-de Jong P., Deinum J. (2012). Hemoglobin level is positively associated with blood pressure in a large cohort of healthy individuals. Hypertension.

[60] Barros E., Manfro R.C., Tomé F.S. (2007). Nefrologia: rotinas, diagnóstico e tratamento.

[61] Griffith T.F., Chua B.S.Y., Allen A.S., Klassen P.S., Reddan D.N., Szczech L.A. (2003). Characteristics of treated hypertension in incident hemodialysis and peritoneal dialysis patients. Am. J. Kidney Dis..

[62] Tapolyai M., Karim J., e Fakhruddin A. (2008). Escalating antihypertensive medications in end-stage renal disease patients does not improve blood pressure control. J. Clin. Hypertens..

[63] Chen R., Lu J., Yu Q., Li P., Yang D., Wang C. (2015). The acute effects of outdoor temperature on blood pressure in a panel of elderly hypertensive patients. Int. J. Biometeorol..

[64] PBMC - Painel Brasileiro de Mudanças Climáticas (2012). Sumario Executivo do Volume 1 – Base Cientifica das Mudanças Climáticas. Contribuição do Grupo de Trabalho 1 para o 1º Relatório de Avaliação Nacional do Painel Brasileiro de Mudanças Climáticas. Volume Especial para a Rio+20. PBMC, Rio de Janeiro, Brasil.

[65] Climate chip. Your area.[cited 29 ago 2017].In: climate chip [Internet]. EUA. http://www.climatechip.org/your-area-tomorrow.

[66] Xu D. (2019). Acute effects of temperature exposure on blood pressure: an hourly level panel study. Environ. Int..

[67] Hommos M., Schinstock C. (2016). Hypertension in the hemodialysis patient. Adv Exp Med Biol. Switzerland.

